# High Mutation Burden in ER-Positive/HER2-Negative/Luminal Breast Cancers

**DOI:** 10.3390/jcm11061605

**Published:** 2022-03-14

**Authors:** Ioannis A. Voutsadakis

**Affiliations:** 1 Algoma District Cancer Program, Sault Area Hospital, Sault Ste. Marie, ON P6B 0A8, Canada; ivoutsadakis@nosm.ca; 2 Section of Internal Medicine, Division of Clinical Sciences, Northern Ontario School of Medicine, Sudbury, ON P3E 2C6, Canada

**Keywords:** mutations, immunotherapy, neoantigens, estrogen receptor, PI3K

## Abstract

Background: Tumor mutation burden (TMB) is arising as a useful marker of checkpoint inhibitors’ effectiveness in cancer patients in general and has been proposed as predictive in breast cancers. Despite the initial success of checkpoint inhibitors in triple-negative breast cancer, ER-positive breast cancers are less amenable to immunotherapy treatments due to the lower immunogenicity of this subset, associated with lower TMB and less pronounced inflammatory cell infiltration. However, a minority of ER-positive breast cancers do have a higher TMB and could be targets of immune checkpoint inhibitors. Methods: This investigation uses publicly available genomic data to examine ER-positive/HER2-negative or luminal breast cancers with high mutation numbers and compare them with cancers of the same subtype and low mutation numbers. Clinical characteristics and molecular correlates according to mutation numbers are described. Results: ER-positive/HER2-negative and luminal breast cancers with high mutation numbers have a higher prevalence of *PIK3CA* mutations and in some of the series examined mutations in *TP53* and *CDH1*. A significant proportion of cancers with high mutation numbers carry mutations in microsatellite instability genes and genes involved in DNA damage response. Despite these differences, the prognosis of ER-positive/HER2-negative and luminal breast cancers with high mutation numbers is not significantly different compared to counterparts with lower mutation counts. Conclusions: These data may inform the potential suitability of these cancers for immunotherapy and could guide the development of rational combination therapies based on immune checkpoint inhibitors with other targeted drugs.

## 1. Introduction

Breast cancer represents the most prevalent female carcinoma [1]. Significant advances in detection, diagnosis, and treatment have improved survival outcomes in the last decades. However, breast cancer remains a leading cause of morbidity and mortality [1]. In addition, when in a metastatic stage, the disease becomes incurable. Breast cancer is not one disease and is classified clinically in at least three subtypes based on the expression of the estrogen receptor (ER) and the human EGFR family receptor 2 (HER2). These subtypes have paramount prognostic and therapeutic implications [2]. Clinical biomarkers used for subtype classification, including ER and HER2 receptors, serve as predictive biomarkers to respective targeted therapies [2]. Besides therapies that inhibit ER and HER2 receptors in the respective subtypes that express them, other targeted therapies are currently in use for the treatment of subsets of breast cancers. Targeted therapies that have improved outcomes in breast cancers and are currently in the clinical armamentarium include CDK kinase inhibitors and PI3K kinase inhibitors in metastatic ER-positive breast cancers, PARP inhibitors for cancers with BRCA1 or BRCA2 mutations, and immune checkpoint inhibitors for triple-negative cancers and cancers with microsatellite instability [3,4,5,6]. Other targeted therapies are in various stages of development, and some of them will undoubtfully find their way to the clinic [7,8].

Initial clinical studies in ER-positive/HER2-negative breast cancers have shown that immunotherapy with immune checkpoint inhibitors produces poor results that are inferior to the results in triple-negative disease [9]. These are partially explained by the lower immunogenicity of ER-positive/HER2-negative breast cancers [10,11,12]. However, a better understanding of the molecular immune environment of this most common subtype of breast cancer could determine less abundant subsets of cases with a genomic landscape producing greater genomic instability that makes them therapeutically amenable to immune therapies [10]. In addition, the immune landscape of these cancers may inform ways that non-immunogenic cancers can be induced to become responsive to immunotherapies through combination treatments. Most promising biomarkers of response to immune checkpoint inhibitors have focused on the expression of the target immune checkpoint protein (in the case of PD-L1/PD-1 inhibitors, expression of PD-L1), the tumor total mutation burden (TMB) as a measurement of neoantigen production, and the ability of the immune system to penetrate the tumor to perform its cytotoxic effects, as measured by the presence of tumor-infiltrating lymphocytes (TILs) [13,14].

This investigation examines ER-positive/HER2-negative breast cancers with high mutation counts in publicly available databases and seeks to derive information on the landscape of these cancers compared with counterparts with lower mutation numbers. Differences in prevalence of mutations and copy number alterations in key pathogenic oncogenes and tumor suppressors in breast cancer are examined. Determination of specific molecular defects in cancers with high versus low mutation counts could inform differences in pathogenesis and could guide therapeutic insights.

## 2. Methods

Published genomic studies of breast cancer were interrogated in the cBioportal platform (http://www.cbioportal.org, accessed on 21 December 2021). cBioportal is a user-friendly publicly available platform containing molecular studies and corresponding clinical data from The Cancer Genome Atlas (TCGA) network and other groups [15,16]. The cBioportal platform empowers a multidimensional interrogation of genomic data, including point mutations, fusions, and copy number alterations. cBioportal links information on molecular lesions in genes included in the original studies with patient clinical characteristics and survival outcomes [15].

The current analysis is based on three studies of breast cancer cohorts, the TCGA, the METABRIC (Molecular Taxonomy of Breast Cancer International Consortium) study, and the metastatic breast cancer cohort from Dana Farber Cancer Institute (DFCI) [17,18,19]. The three studies used different genomic platforms. The TCGA breast cancer study presented data from a whole-exome evaluation, while the two other studies used targeted genomic panels. The METABRIC study used a targeted panel of 173 genes [20]. The DFCI study used 2 different platforms called Oncopanel 1, consisting of 85 genes, and Oncopanel 2, consisting of 351 genes [19]. Copy number alterations (CNAs) analysis was performed in the included studies with the GISTIC (Genomic Identification of Significant Targets in Cancer) algorithm [21]. GISTIC uses Affymetrix DNA microarray files to infer the copy number of different regions of the genome, allowing for calculation of copy number of individual gene loci. Putative amplification of a given gene is defined in the algorithm as a score of 2 or above. The original calculations of putative copy numbers as provided in cBioportal have been used. The TCGA study provides Aneuploidy Score (AS) as a measure of chromosomal instability of each sample. AS is calculated as the sum of the number of chromosome arms in each sample that have copy number alterations (gains or losses). A chromosome arm is considered copy number altered, either gained or lost, if there is a somatic copy number alteration in more than 80% of the length of the arm as calculated by the ABSOLUTE algorithm, which is based on Affymetrix 6.0 SNP arrays [22]. Chromosomal arms with somatic copy number alterations in 20–80% of the arm length are considered not evaluable and chromosomal arms with somatic copy number alterations in less than 20% of the arm length are considered not altered.

Definition of high mutation number groups varied to take into consideration the differences between the genomic platforms in the three interrogated studies. For the TCGA study, the high TMB group included patients with TMB above 10 non-synonymous mutations per MB. The cut-off for the two other studies using targeted genomic platforms was set to 10 total number of mutations per sample.

The Fisher’s exact test or the x^2^ test and the *t*-test, respectively, are used to compare categorical and continuous data. Kaplan–Meier survival curves were constructed and compared using the Log Rank test. All statistical comparisons were considered significant if *p* < 0.05.

No external funding was received from any source for the performance of this study.

## 3. Results

Among the 1084 patients in the breast cancer cohort from TCGA, 696 patients (64.2%) had luminal cancers (luminal A and luminal B) and are included in the current analysis. Eighteen patients with luminal breast cancers (2.6%) had a TMB above 10, and 678 patients (97.4%) had a TMB of 10 or below. Due to the small number of patients in the high TMB group, some of the statistical calculations may be underpowered for confirming statistically significant differences. Characteristics of the whole group of TCGA patients with luminal breast cancers and the two groups with high (above 10) TMB and low (≤10) TMB are shown in Table 1. Almost all patients in the series had localized disease and had not received any neoadjuvant therapy before their surgery. No significant differences in the age, stage, or histologic type of breast cancer were observed between the groups with low and high TMB (Table 1). Adjuvant radiation was used in a higher percentage of patients in the low TMB group (Fisher’s exact test *p* = 0.04).

**Figure 1:**
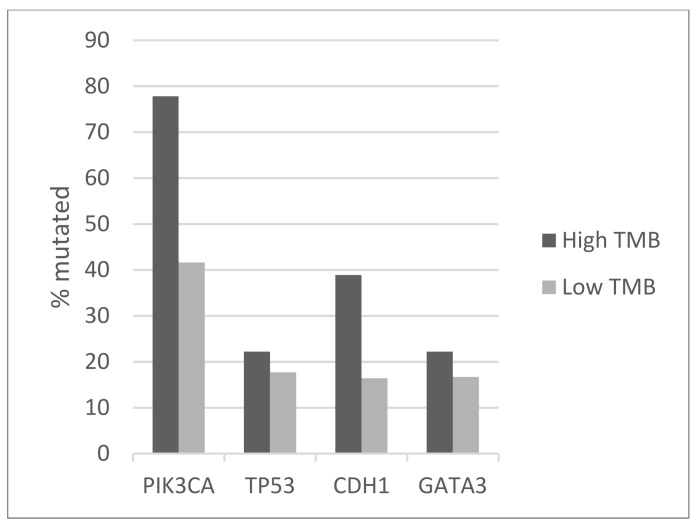
Prevalence of mutations in frequently mutated genes *PIK3CA*, *TP53*, *CDH1*, and *GATA3* in luminal breast cancers according to TMB. High TMB group: >10 mutations/MB; low TMB group: ≤10 mutation/MB. The prevalence difference between the groups for *PIK3CA* and *CDH1* mutations is statistically significant (Fisher’s exact test *p* = 0.003 and 0.02, respectively). The prevalence difference between the groups for *TP53* and *GATA3* mutations is not statistically significant (Fisher’s exact test *p* = 0.54 and 0.52, respectively). Data are from TCGA. TMB—tumor mutation burden.

**Figure 2:**
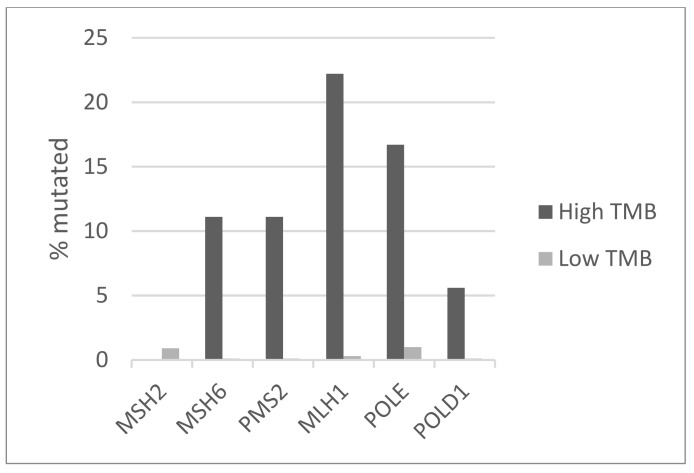
Prevalence of mutations in mismatch repair-related genes and genes encoding for polymerases *POLE* and *POLD1* in luminal breast cancers according to TMB. The total non-overlapping prevalence of these mutations in the high TMB group is 44.4% and in the low TMB group is 2.6% (Fisher’s exact test *p* < 0.000). High TMB group: >10 mutations/MB; low TMB group: ≤10 mutation/MB. Data are from TCGA. TMB—tumor mutation burden.

**Figure 3:**
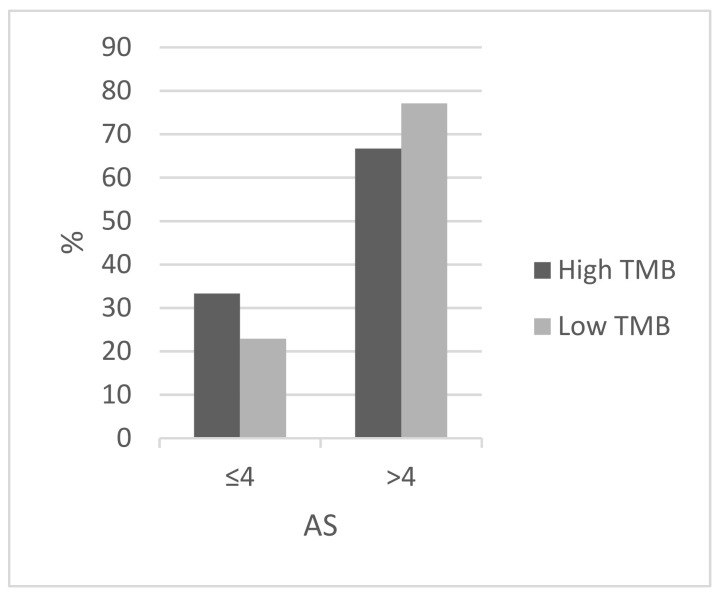
Prevalence of Aneuploidy Score (AS) of above or below 4 in luminal breast cancers according to TMB (Fisher’s exact test *p* = 0.39). High TMB group: >10 mutations/MB, low TMB group: ≤10 mutation/MB. Data are from TCGA. TMB—tumor mutation burden.

The most frequently mutated genes in the luminal breast cancer TCGA cohort included *PIK3CA*, *TP53*, *CDH1*, and *GATA3* mutated in 42.5%, 17.8%, 16.9%, and 16.8% of cases, respectively. Among those, statistically significant differences in prevalence between the high and low TMB groups were observed for *PIK3CA* and *CDH1*, which were mutated in 77.8% and 38.9% of cases in the high TMB group versus 41.6% and 16.4% of cases in the low TMB group (Fisher’s exact test *p* = 0.003 and 0.02, respectively, Figure 1). In contrast, mutations in *TP53* and *GATA3* had smaller differences in prevalence in the two groups. Patients with luminal cancers and a high TMB displayed a higher prevalence of mutations in MSI-related genes and genes encoding for the proofreading polymerases epsilon, *POLE* and delta, *POLD1* compared with the group with low TMB (Figure 2). Mutations in *MSH6*, *MLH1*, and *PMS2* were observed in 11.1%, 11.1%, and 22.2% of cases in the high TMB group, compared with 0.1%, 0.1%, and 0.3% in the group with low TMB. Mutation in *POLE* and *POLD1* were observed in 16.7% and 5.6% of patients in the high TMB group compared with 1% and 0.1% of patients with low TMB. Overall, 44.4% of patients with luminal breast cancers in the high TMB group display at least one MSI-associated or proofreading polymerase mutation compared with 2.6% of patients in the low TMB group (Fisher’s exact test *p* = 0.0001). Mutations in DNA damage response genes such as *BRCA1* and *BRCA2* are also observed more frequently in patients with high TMB, while they are rare in the low TMB group (Table 2). *BRCA1* was mutated in four patients (22.2%) in the high TMB group. Albeit individually rare, mutations in DNA damage response genes as a group are common in high TMB cancers and statistically significantly more prevalent than in luminal cancers with low TMB (Fisher’s exact test *p* = 0.0001). Genes encoding for proteins of the PI3K/AKT/mTOR pathway and for receptor tyrosine kinases were more frequently mutated in patients with high TMB. For example, the tumor suppressor phosphatase PTEN showed mutations in 44.4% of samples in the high TMB group and in 4.9% of samples in the low TMB group (Table 2). In addition, mutations in the *ERBB2* gene encoding for HER2 receptor are observed in 27.8% of samples with high TMB and 2.1% of those with low TMB. Chromosomal instability as measured by an Aneuploidy Score above 4 was more frequently observed in the low TMB group (77.1% of patients) than in the high TMB group (66.7% of patients, Figure 3), but the difference did not reach statistical significance (Fisher’s exact test *p* = 0.39). Similar results were observed for another measure of chromosomal instability, the fraction of genome altered. Regarding copy number alterations, specific genes that were differentially altered in the high and low TMB groups included *BRIP1* at locus 17q23.2, which was amplified in 5 patients (27.8%) from the high TMB group and in 57 patients (8.4%) in the low TMB group (Fisher’s exact test *p* = 0.01, Table 3). However, the neighboring gene *ERBB2* at 17q12 was amplified in similar percentages in the two groups (11.1% and 7.8%). *CCND1* encoding for cyclin D1 was also amplified in similar frequencies in the two groups (22.2% and 19.5% in the high TMB and the low TMB group, respectively). The amplicon at 8p11.23 that includes *NSD3* and *FGFR1* was amplified in 12.7% of patients in the low TMB group and in none of the high TMB group. Amplifications of oncogene *MYC* were observed only in cases with low TMB (Table 3). With the caveat of small numbers in the high TMB group, survival comparison analysis showed that the high and low TMB groups had similar overall survival, disease-specific survival, and disease-free survival (Log Rank p 0.9, 0.5, and 0.6, respectively, not shown).

In the METABRIC cohort of 2509 patients with mostly localized breast cancers, 1301 patients (51.9%) had ER-positive/HER2-negative breast cancers and were included in the analysis. Of the 1301 patients, 97 patients (7.5%) had mutation counts above 10 and were classified in the high mutation count group, and 1204 patients (92.5%) had mutation counts of 10 or below and were included in the low mutation count group (Table 4). The two groups did not differ significantly in the mean age of patients, the inferred menopause status, the stage, or the histologic type of tumors. The group with high mutation counts had more frequently (43.3%) high-grade tumors compared with the low mutation count group that had high-grade tumors in 35.5% of cases (Table 4, Fisher’s exact test *p* = 0.04). No significant differences between the groups were observed in PR expression and the molecular classification according to the 3 gene classifier or the PAM50 category. The integrative cluster classification also showed a balanced distribution in the two groups, with clusters 3, 8, 4ER+, and 7 comprising collectively more than three-fourths of cases in both groups. The low mutation group received more frequent adjuvant treatments, including hormonal therapy, radiation therapy, and chemotherapy, although only a small percentage of patients in both groups received chemotherapy (Table 4).

**Figure 4:**
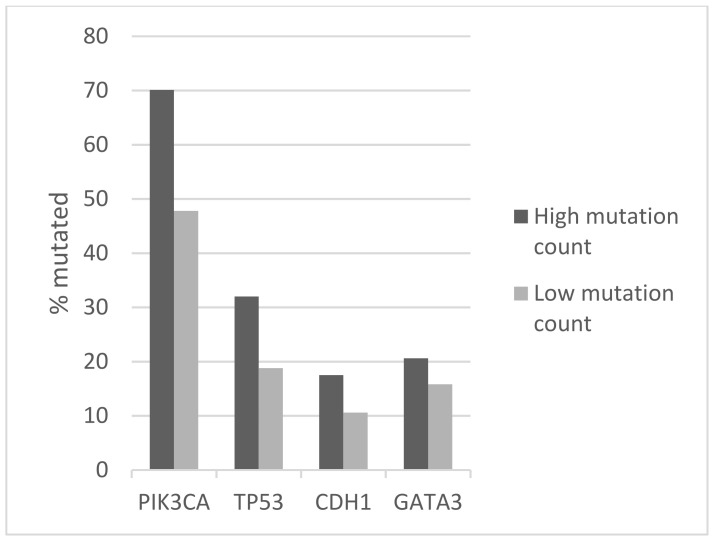
Prevalence of mutations in frequently mutated genes *PIK3CA*, *TP53*, *CDH1*, and *GATA3* in ER-positive/HER2-negative breast cancers according to total mutation count. The prevalence difference between the groups for *PIK3CA*, *TP53*, and *CDH1* mutations is statistically significant (Fisher’s exact test *p* < 0.0001, 0.003, and 0.04, respectively). The prevalence difference between the groups for *GATA3* mutations is not statistically significant (Fisher’s exact test *p* = 0.24). High mutation count group: >10 mutations/MB, low mutation count group: ≤10 mutation/MB. Data are from the METABRIC study.

**Figure 5:**
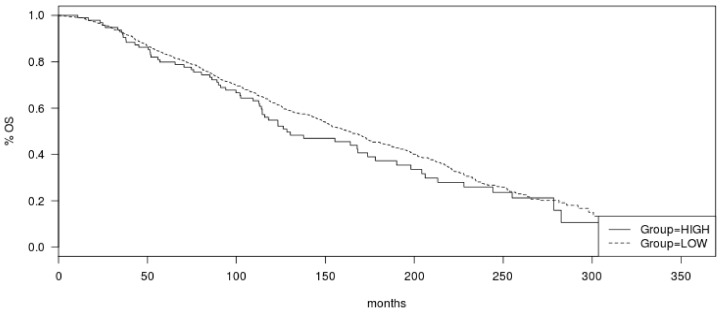
Overall survival (OS) of ER-positive/HER2-negative breast cancers according to total mutation count in the METABRIC cohort. Log Rank *p* = 0.28.

**Figure 6:**
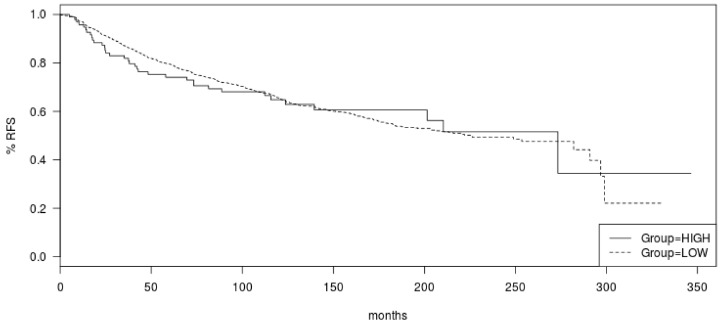
Relapse-free survival (RFS) of ER-positive/HER2-negative breast cancers according to total mutation count in the METABRIC cohort. Log Rank *p* = 0.9.

The commonly mutated oncogenes and tumor suppressor genes in luminal breast cancers, including oncogenic kinase *PIK3CA* and tumor suppressors *TP53* and *CDH1*, were more frequently mutated in tumors with high mutation numbers in the METABRIC cohort (Figure 4). *PIK3CA* mutations were observed in 70.1% of cases in the high mutation group and in 47.8 of cases in the low mutation count group (Fisher’s exact test *p* < 0.0001). Similarly, *TP53* mutations and *CDH1* mutations occurred in 32% and 17.5% of cases in the high mutation number group and in 18.8% and 10.6% of cases in the low mutation count group (Fisher’s exact test *p* = 0.003 and 0.04, respectively). Mutations in transcription factor *GATA3* were also more common in the high mutation count group (20.6% versus 15.8% in the low mutation count group), but this difference did not reach statistical significance (*p* = 0.24, Figure 4). Besides *TP53*, mutations in other genes involved in the DNA damage response and repair, such as *BRCA1*, *BRCA2*, and *BRIP1*, were more frequently observed in the high mutation count group. Collectively, 20.6% of cases in the high mutation count group had mutations in *BRCA1*, *BRCA2*, *BRIP1*, and *ATR*, while the aggregate prevalence of these mutations in the low mutation count group was 6.1% (Fisher’s exact test *p* < 0.0001, Table 5). Mutations of the PI3K pathway, besides *PIK3CA* and mutations of EGFR receptor tyrosine kinase members, were also significantly more frequent in the high mutation count group (Table 5). Regarding copy number alterations, amplifications of *CCND1* and amplifications of genes at the 17q amplicon had similar prevalence in the high and low mutation count groups (Table 3). Consistent with the data from TCGA, *FGFR1* and *MYC* amplifications were more common in the low mutation count group. Similar to the TCGA cohort, survival analysis of the METABRIC cohort of ER-positive/HER2-negative breast cancers showed no significant differences in OS and RFS between the high and low mutation count groups (Log Rank p 0.28 and 0.9, respectively, Figure 5 and Figure 6).

**Figure 7:**
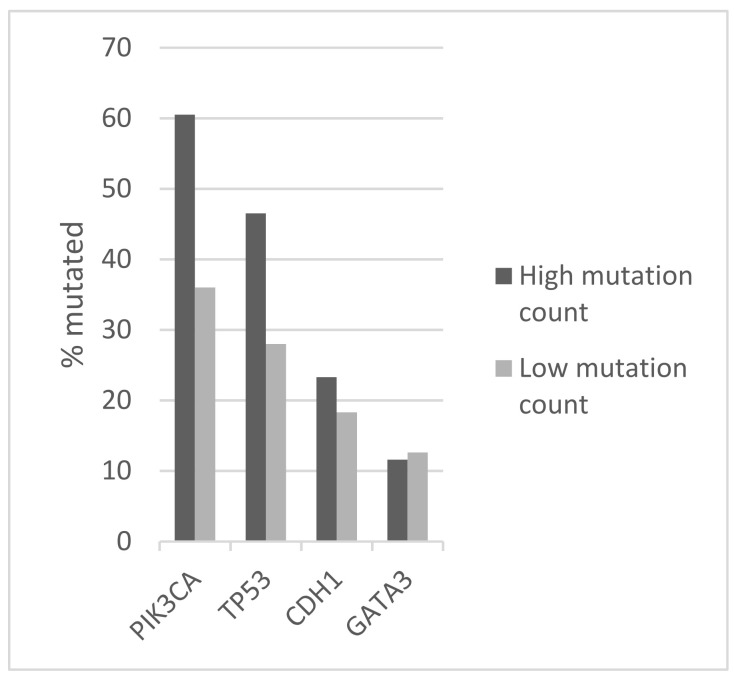
Prevalence of mutations in frequently mutated genes *PIK3CA*, *TP53*, *CDH1*, and *GATA3* in ER-positive/HER2-negative breast cancers according to total mutation count. The prevalence difference between the groups for *PIK3CA* and *TP53* mutations is statistically significant (Fisher’s exact test *p* = 0.002 and 0.01, respectively). The prevalence difference between the groups for *CDH1* and *GATA3* mutations is not statistically significant (Fisher’s exact test *p* = 0.4 and 0.99, respectively). High mutation count group > 10 mutations/MB; low mutation count group ≤ 10 mutation/MB. Data are from the DFCI metastatic breast cancer study cohort.

**Figure 8:**
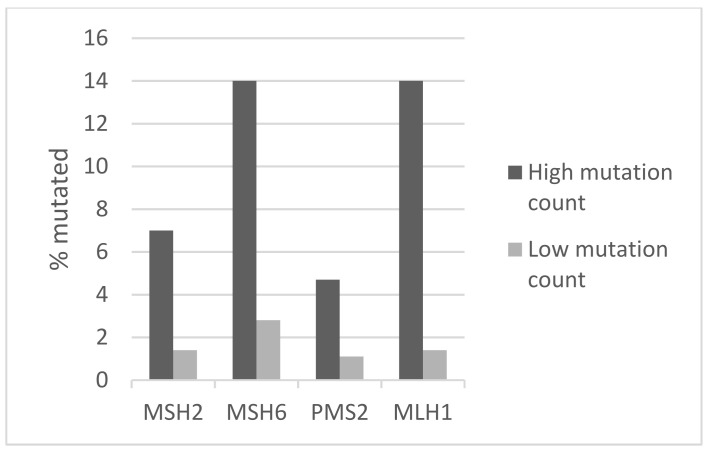
Prevalence of mutations in mismatch repair-related genes in ER-positive/HER2-negative breast cancers according to total mutation count (Fisher’s exact test for the comparison of total non-overlapping mutations in the 4 genes between the 2 groups *p* < 0.000). High mutation count group > 10 mutations/MB; low mutation count group ≤ 10 mutation/MB. Data are from the DFCI metastatic breast cancer study cohort.

**Figure 9:**
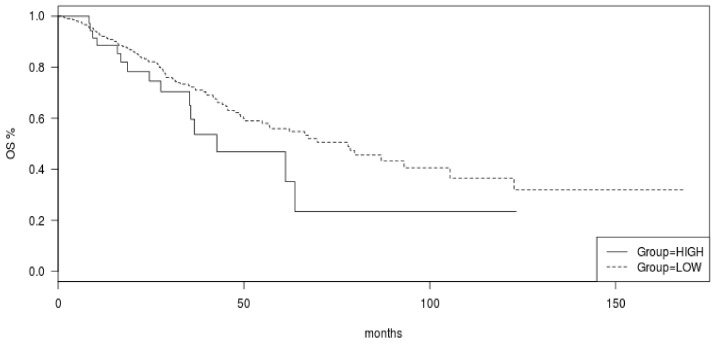
Overall survival (OS) of ER-positive/HER2-negative breast cancers according to total mutation count in the DFCI metastatic breast cancer cohort. Log Rank *p* = 0.15.

In the genomic study of 856 metastatic breast cancer patients from DFCI, tumor samples were examined with the use of the Oncopanel 1 and Oncopanel 2 targeted gene panels. Among the analyzed cohort, 480 patients (55.9%) had ER-positive/HER2-negative cancers at diagnosis and were included in the current analysis. The percentage of ER-positive/HER2-negative patients who had a mutation count of more than 10 mutations was 8.95% (Table 6). The mean mutation number in these patients was 15.95 (SD: 8), while the mean mutation number in the group of patients with a mutation count of 10 or fewer mutations was 5.49 (SD: 2.27). Among the characteristics of the two groups, there were no significant differences in the age of metastatic cancer diagnosis, initial stage, histologic type, tumor grade, time to recurrence, subtype of metastatic disease, and previous adjuvant treatments received (Table 6). Consistent with the two other series, most common mutations in genes examined in the Oncopanels in the whole ER-positive/HER2-negative population included mutations in *PIK3CA* observed in 38.1% of cases, mutations in *TP53* in 29.6% of cases, mutations in *CDH1* in 18.8% of cases, and mutations in *GATA3* prevalent in 12.5% of cases. Mutations in *PIK3CA* were present in the majority of cases with a high mutation count, observed in 60.5% of cases. TP53 mutations, *CDH1* mutations, and *GATA3* mutations were present in 46.5%, 23.3%, and 11.6% of cases, respectively. In the low mutation count group, mutations in *PIK3CA* and *TP53* were observed in a significantly lower percentage of cases (36% and 28%, Fisher’s exact test p 0.002 and 0.01, respectively, Figure 7). In contrast, mutation rates of *CDH1* and *GATA3* were not statistically different between the high and low mutation count groups. The group of ER-positive/HER2-negative patients with high mutation count had a higher prevalence of mutations of genes associated with microsatellite instability, *MSH2*, *MSH6*, *MLH1*, and *PMS2* observed in 7%, 14%, 14%, and 4.7% of cases, compared with 1.4%, 2.8%, 1.4%, and 1.1% of cases in the low mutation count group (Figure 8). Other mutations enriched in the high mutation group with putative therapeutic implications include mutations in genes of DNA damage response, other genes of the PI3K/AKT/mTOR pathway besides *PIK3CA*, and genes encoded for receptor tyrosine kinases (not shown). These genes have individually low frequencies of mutations but as groups account for a significant minority of cases with high mutation counts. Regarding copy number alterations, the targeted panels used in the DFCI series confirmed that the most frequent amplifications in both high and low mutations groups were at the loci of *CCND1*, encoded for cyclin D1 at chromosome 11q13.3 and of *FGFR1* at chromosome 8p11.23 (Table 3). In general, common amplifications were not very different in the high and low mutations group. In contrast to the two other series, *FGFR1* amplifications at 8p11.23 were more equally distributed between the two groups in the DFCI cohort (Table 3). Similar to the two other series with localized disease, the OS of the two groups with high and low mutation counts in the DFCI series with metastatic ER-positive/HER2-negative cancers were not different (Log Rank *p* = 0.15, Figure 9).

## 4. Discussion

The burden of mutations carried in a tumor has potential implications for its behavior and response to therapies [23]. This has become more evident with the introduction of immunotherapies in the form of immune checkpoint inhibitors in clinical oncology therapeutics. These treatments tend to be more efficacious in a tumor molecular environment with a high number of mutations [24]. Indeed, one of these drugs, pembrolizumab, has been granted approval in tumors with a high TMB agnostic of the primary site [25,26].

Luminal breast cancers have not been among the cancers that are amenable to immune treatments, and responses to treatment with immune checkpoint inhibitors are rarely observed in the clinical trials contacted so far [9,27]. However, there may exist subsets of luminal breast cancers that could respond better to immune therapies and derive benefit from these therapies that have provided an efficacious and acceptably well-tolerated option in other cancers. Besides the expression of the ligand of the targeted ligand–receptor couple, PD-L1, the mutation number that the tumor harbors is the most obvious potential candidate biomarker of response to these drugs. The total number of cancer cell mutations is a source of neoantigens for presentation to the immune cells that are activated by the drugs [28]. Thus, the current investigation describes the landscape of ER-positive/HER2-negative breast cancers or the similar, albeit not completely overlapping, genomic subset of luminal cancers, as it pertains to their total mutation number. This research relies on previously published genomic data and does not attempt to reproduce or experimentally validate these data. Instead, it attempts to derive useful patterns that may provide clinical clues of pathogenicity and directions for therapeutic developments. The three large publicly available series used in this investigation comprise a total of 2800 breast cancer patients with these overlapping phenotypes and cover both localized and metastatic patients. They differ in the genomic platform used, with the METABRIC and DFCI studies performing the genomic analysis with a targeted panel of 173 and 351 genes, respectively. A subset of cases in the DFCI study was evaluated with a smaller panel of 85 genes. TCGA performed a comprehensive whole-exome sequencing. Due to the different genomic platforms used, the criteria for inclusion in the high mutation count groups differed in the three cohorts, a fact that may explain the different prevalence of high mutation counts that was 2.6%, 7.5%, and 8.95% in TCGA, METABRIC, and DFCI, respectively. Overestimation of mutation burden from targeted panels has been described previously and contributes to the higher percentage of the high mutation groups in the METABRIC and DFCI cohorts [29]. In addition, patients from the two targeted studies were selected based on ER-positive/HER2-negative status by immunohistochemistry, while this classification was not available in TCGA, and patients were selected based on the genomic luminal classification. Despite differences between the examined series, common themes in the mutation count landscape of ER-positive/HER2-negative luminal breast cancers are confirmed (Table 7). The gene encoding for the catalytic sub-unit alpha of PI3K kinase, *PIK3CA*, is the most frequently mutated oncogene in ER-positive/HER2-negative/luminal cancers in all three cohorts and is significantly enriched in tumors with high mutation counts. TP53 and CDH1 are also more frequently mutated in cancers with high mutation numbers, but the difference was significant in two of the three cohorts. Differences in attaining significance may partially stem from the low number of patients in the high TMB group of TCGA, which decreases the statistical power of the comparisons in this cohort. In addition, data from both TCGA and DFCI cohorts (the panel used in METABRIC does not evaluate these genes) show that genes involved in mismatch repair and encoding for proofreading polymerases epsilon and delta are also more common in the high mutations group, and although individually uncommon, are cumulatively present in a significant percentage of these cases. These observations may imply that mutations in any of those genes contribute to the development of genomic instability and increase in the total mutation number. An alternative hypothesis would suggest that increased mutation numbers developing in these cancers are caused by increased genomic instability due to alternative pathogenic factors. In that case, common mutations observed in oncogenes, tumor suppressors, MMR-associated and polymerase genes would be random passengers. The latter hypothesis is plausible and would result in mutations developing across the length of these target genes. However, this is not the case. Rather, mutations in the *PIK3CA* gene, for example, tend to aggregate in a non-random fashion to the hotspot amino acid positions E543, E545, and H1047, which are known pathogenic. This fact strongly suggests that activating mutations in these hotspot amino acid positions are causally related to genomic instability and the pathogenesis of high total mutation numbers in ER-positive/HER2-negative/luminal breast cancers. A similar association of hotspot *PIK3CA* mutations with a high tumor mutation burden has been described in colorectal cancers [30]. Increased prevalence of function-affecting mutations in tumor suppressor *TP53*, in DDR-related genes, and in genes encoded for other proteins of the PI3K/AKT pathway besides PIK3CA may be causally involved in the pathogenesis of additional cases of ER-positive/HER2-negative breast cancers with high mutation counts.

The underlying process that leads to the hypermutated phenotype is critical for the understanding of the pathogenesis of hypermutated cancers and may also have therapeutic implications as the type and antigenicity of mutations produced with each process is distinct [31,32]. As a result, the same number of total mutations in a tumor may not result in the same responsiveness to immune therapies. A study of mutation signatures across breast cancers has shown that the most prevalent signature in hypermutated breast cancers is related to deaminases of the APOBEC family [29]. Lower prevalence was observed for homologous recombination signature, MMR, and proofreading polymerases signature. APOBEC deaminase overexpression is observed in breast cancers with *PIK3CA*, *TP53* mutations, and DNA damage response activating ATR [33,34]. Thus, based on these associations, the increased prevalence of these molecular abnormalities in ER-positive/HER2-negative/luminal breast cancers may underline an APOBEC mutational signature. Response to the checkpoint inhibitor pembrolizumab was shown in a few cases with APOBEC and MMR-associated hypermutated breast cancers [29]. However, even the same mutation signature does not lead to uniform responsiveness to immunotherapy, and variation of the produced total mutation number may still be critical, as shown in the variability of response to pembrolizumab in endometrial cancers with mismatch repair due to Lynch syndrome as compared with sporadic mismatch repair defects [35].

Despite an increasing number of mutations that suggest increased immunogenicity and improved outcomes, survival evaluations in all three cohorts show that a high mutation count is not associated with improved prognosis in either localized or metastatic cancers. Although data presented here solidly confirm that ER-positive/HER2-negative/luminal breast cancers with high and low tumor mutation numbers have similar prognoses, they do not inform regarding total tumor mutation numbers as predictive markers of immunotherapy response, as the patients in the series examined did not receive immunotherapies. The lack of prognostic implications of mutation numbers is expected in metastatic disease, as the presence of metastases implies escape from immune surveillance and has been described in other cancers [36]. Thus, factors in the tumor microenvironment of these cancers prevent the immune system from mounting an effective immune response. This is consistent with observations from clinical trials that have shown low response rates in ER-positive/HER2-negative breast cancers [9,37]. In an early phase trial of pembrolizumab in 25 ER-positive/HER2-negative metastatic breast cancer patients with a combined positive score for PD-L1 of 1% or more, the response rate was 12%, and the median PFS was 1.8 months [9]. Most patients had received multiple lines of therapy in the metastatic setting. The total mutation count was not an inclusion criterion in this study, and the chosen cut-off for PD-L1 may have been sub-optimal in this population.

Data from the TAPUR phase 1/2 trial of pembrolizumab in metastatic breast cancers of any subtype with TMB above 9 showed a response rate of 21% and median PFS of about 2.5 months [37]. Combination immunotherapies could improve the response to monotherapies by activating the cold immune microenvironment of luminal breast cancers in multiple nodes, as observed in other cancers [38]. A phase 2 trial of nivolumab with the CTLA-4 inhibitor ipilimumab (NIMBUS) in HER2-negative breast cancer patients selected for a high TMB above 9 reported a response rate of 16% and suggested that patients with higher TMB above 14 may achieve much higher responses of 60% [39]. This trial included both ER-positive and triple-negative patients. Thus, whether the combination approach offers any incremental benefit in this population is unclear. The optimal cut-off of total mutation number in ER-positive/HER2-negative breast cancers as a biomarker of immunotherapy response also remains to be determined and may depend on the specific immunotherapy drug or combination. However, from the response rates with PD-1 monotherapies, it is evident that the total mutation count in a tumor is an imperfect biomarker for immune response, and other factors are required for an effective antitumor immune response. Antigen presentation is such a required step for immune cells to be able to sense the mutations and be activated. Indeed, mutations in antigen presentation machinery are a described mechanism of tumors with a high mutation burden to avoid immune detection and destruction [40].

The number and type of tumor-infiltrating lymphocytes (TILs) and other immune cells present in the tumor microenvironment are also relevant for an immune antitumor response. Tumors are categorized as hot when a high number of TILs are present inside the tumor stroma, immune excluded when immune cells are present in the periphery of the tumor but not penetrating between the tumor cells, and cold when immune cells are completely absent [41]. A high number of TILs has been associated with a better prognosis and response to immune therapies [42]. In contrast to other breast cancer subtypes (triple-negative and HER2-positive) where TILs are associated with decreased mutation number, believed to be due to tumor immunoediting of clones with high mutation burden, in ER-positive cancers, total mutation number is significantly associated with the presence of TILs [43]. This suggests that in a subset of ER-positive cancers, the tumor microenvironment prevents TILs that tend to be attracted by cancer cells with a higher number of mutations from attacking them. Moreover, it presents a therapeutic opportunity for immunotherapy if these factors could be identified and negated. Targeted therapies inhibiting pathways that have been shown in the current report to segregate with high mutation numbers, such as PI3K inhibitors and PARP inhibitors (most effective in cancer cells with homologous recombination deficiency), are candidates for development in combination with immune checkpoint inhibitors to sensitize ER-positive/HER2-negative breast cancers to immunotherapies and improve the mediocre results obtained in previous clinical trials. Similar pathways of development are already explored in other cancers with low immunogenicity, such as ovarian cancers and endometrial cancers with MMR proficiency [44].

## 5. Conclusions

A minority of ER-positive/HER2-negative/luminal breast cancers harbor a high TMB and display a constellation of associated molecular defects described in the preceding sections. These defects are not homogeneously present in all high TMB ER-positive/HER2-negative/luminal breast cancers but rather characterize subsets of these cancers that need to be specifically sought through molecular investigations. Targeted treatments combining immunotherapies with inhibitors of additional oncogenic pathways could be more effective in the subsets harboring the specific target lesions. Therapeutic combinations will have a higher chance of successful development against specific cancers that harbor both high TMB, increasing the chances of immune checkpoint inhibitors’ effectiveness and the target lesions of the drug(s) that is combined with them, such as PI3K inhibitors for cancers with *PIK3CA* mutations or PARP inhibitors for cancer with homologous recombination defects.

## Figures and Tables

**Table 1:** Characteristics of the high and low TMB groups in patients with luminal cancers from the breast cancer TCGA study. In parentheses are percentages. Stage was not available in 13 patients. IDC—invasive ductal carcinoma; ILC—invasive lobular carcinoma; TMB—tumor mutation burden; NA—not available.

Characteristic	Whole Group (*n* = 696)	High TMB (*n* = 18) (%)	Low TMB (*n*= 678) (%)	*p*
Age at diagnosis (mean ± SD)	58.5 ± 16.1	58.4 ± 16.7	59.2 ± 13.3	0.8
Age				
>50 years old	500 (71.8)	12 (66.7)	488 (72)	0.6
≤50 years old	196 (18.2)	6 (33.3)	190 (28)	
Stage at diagnosis				
I	131 (18.8)	3 (16.7)	128 (18.9)	0.29
II	374 (53.6)	13 (72.2)	361 (53.2)	
III	167 (24)	2 (11.1)	165 (24.3)	
IV	11 (1.6)	0	11 (1.6)	
Histology				
IDC	484 (69.5)	10 (55.6)	474 (69.9)	0.32
ILC	145 (20.8)	7 (38.9)	138 (20.4)	
Mixed	22 (3.2)	0	22 (3.2)	
Other	45 (6.5)	1 (5.5)	44 (6.5)	
Radiation therapy				
Yes	351 (50.4)	5 (27.8)	346 (51)	0.04
No	285 (40.9)	12 (66.7)	273 (40.3)	
NA	60 (8.6)	1 (5.5)	59 (8.7)	

**Table 2:** Mutated genes with possible therapeutic interest in the high (*n* = 18) and low mutation (*n* = 678) count groups in patients with luminal cancers from the breast cancer TCGA study. In parentheses are percentages.

Gene	High Mutation Count (%)	Low Mutation Count (%)
DNA damage response		
BRCA1	4 (22.2)	8 (1.2)
BRCA2	3 (16.7)	10 (1.5)
PALB2	1 (5.6)	1 (0.1)
BRIP1	1 (5.6)	6 (0.9)
ATM	3 (16.7)	15 (2.2)
POLQ	2 (11.1)	7 (1)
Total (non-overlapping)	10 (55.6)	44 (6.5)
PI3K/AKT/mTOR pathway		
PIK3C2B	3 (16.7)	4 (0.6)
PIK3R1	3 (16.7)	14 (2.1)
PTEN	8 (44.4)	33 (4.9)
AKT1	0	26 (3.8)
TSC1	1 (5.6)	5 (0.7)
TSC2	2 (11.1)	3 (0.4)
MTOR	5 (27.8)	11 (1.6)
Total (non-overlapping)	14 (77.8)	92 (13.6)
Receptor tyrosine kinases		
EGFR	0	7 (1)
ERBB2	5 (27.8)	14 (2.1)
ERBB3	3 (16.7)	5 (0.7)
FGFR1	2 (11.1)	4 (0.6)
FGFR3	1 (5.6)	0
FGFR4	1 (5.6)	0
NTRK1	1 (5.6)	3 (0.4)
NTRK2	0	7 (1)
NTRK3	2 (11.1)	3 (0.4)
PDGFRA	2 (11.1)	1 (0.1)
PDGFRB	4 (22.2)	4 (0.6)
RET	3 (16.7)	3 (0.4)
Total (non-overlapping)	10 (55.6)	51 (7.5)

**Table 3:** Amplifications commonly observed in the three series according to TMB/mutation count groups. In parentheses are percentages.

Gene	Locus	TCGA		METABRIC		DFCI	
		High TMB	Low TMB	High Mutation Count	Low Mutation Count	High Mutation Count	Low Mutation Count
CCND1	11q13.3	4 (22.2)	132 (19.5)	19 (19.6)	239 (19.9)	6 (14)	76 (17.4)
ERBB2	17q12	2 (11.1)	53 (7.8)	1 (1)	45 (3.7)	0	3 (0.7)
BRIP1	17q23.2	5 (27.8)	57 (8.4)	8 (8.2)	105 (8.7)	2 (4.7)	13 (3)
FGFR1	8p11.23	0	86 (12.7)	8 (8.2)	185 (15.4)	4 (9.3)	51 (11.7)
MDM4	1q32.1	1 (5.6)	71 (10.5)	26 (26.8)	348 (28.9)	0	2 (0.5)
MYC	8q24.1	0	68 (10)	13 (13.4)	267 (22.2)	1 (2.3)	25 (5.7)

**Table 4:** Characteristics of the high and low mutation count groups in patients with ER-positive/HER2-negative breast cancer patients from the METABRIC cohort. DCIS—ductal carcinoma in situ; IDC—invasive ductal carcinoma; ILC—invasive lobular carcinoma; NA—not available. In parentheses are percentages.

Characteristic	Whole Group	High Mutation Count (*n* = 97)	Low Mutation Count (*n* = 1204)	*p*
Age at metastasis (mean ± SD)	63.5 ± 12	63.9 ± 11.2	63.3 ± 12	0.6
Inferred menopause status				
Post-menopausal	1099 (84.5)	86 (88.7)	1013 (84.1)	0.3
Pre-menopausal	202 (15.5)	11 (11.3)	191 (15.9)	
Stage at diagnosis				
DCIS	1 (0.07)	0	1 (0.1)	0.46
I	346 (26.6)	26 (26.8)	320 (26.6)	
II	546 (42)	35 (36.1)	511 (42.4)	
III	60 (4.6)	2 (2.1)	58 (4.8)	
IV	7 (0.5)	1 (1)	6 (0.5)	
NA	341 (26.2)	33 (34)	308 (25.6)	
Histology				
IDC	968 (74.3)	68 (70.1)	900 (74.8)	0.53
ILC	117 (9)	12 (12.4)	105 (8.7)	
Mixed	179 (13.7)	13 (13.4)	166 (13.8)	
Other	39 (3)	4 (4.1)	33 (2.7)	
Grade				
I	148 (11.4)	4 (4.1)	144 (12)	0.04
II	627 (48.2)	47 (48.5)	580 (48.2)	
III	470 (36.1)	42 (43.3)	428 (35.5)	
NA	56 (4.3)	4 (4.1)	52 (4.3)	
PR				
Positive	886 (68.1)	60 (61.9)	826 (68.6)	0.17
Negative	415 (31.9)	37 (38.1)	378 (31.4)	
3 Gene classifier				
ER+/HER2 negative Prolif low	568 (43.6)	31 (32)	537 (44.6)	0.06
ER+/HER2 negative Prolif high	547 (42)	52 (53.6)	495 (41.1)	
ER−/HER2−	47 (3.6)	5 (5.2)	42 (3.5)	
HER2+	10 (0.8)	0	10 (0.8)	
NA	129 (9.9)	9 (9.3)	120 (10)	
PAM50				0.3
Luminal A	630 (48.4)	43 (44.3)	587 (48.8)	(Luminal vs. other)
Luminal B	392 (30.1)	29 (29.9)	363 (30.1)	
HER2	69 (5.3)	13 (13.4)	56 (4.7)	
Normal	102 (7.8)	4 (4.1)	98 (8.1)	
Basal	26 (2)	5 (5.2)	21 (1.7)	
Claudin-low	76 (5.8)	2 (2.1)	74 (6.1)	
Not classifiable	6 (0.5)	1 (1)	5 (0.4)	
Adjuvant hormonal therapy				
yes	944 (72.6)	57 (58.8)	887 (73.7)	0.002
no	357 (27.4)	40 (41.2)	317 (26.3)	
Adjuvant chemotherapy				
yes	111 (8.5)	2 (2.1)	109 (9.1)	0.01
no	1190 (91.5)	95 (97.9)	1095 (90.9)	
Adjuvant radiation therapy				
yes	745 (57.3)	41 (42.3)	704 (58.5)	0.002
no	556 (42.7)	56 (57.7)	500 (41.5)	

**Table 5:** Mutated genes with therapeutic interest in the high and low mutation count groups in ER-positive/HER2-negative breast cancer patients from METABRIC. In parentheses are percentages.

Gene	High Mutation Count (%) (*n* = 97)	Low Mutation Count (%) (*n* = 1204)
DNA damage response		
BRCA1	6 (6.2)	11 (0.9)
BRCA2	5 (5.2)	17 (1.4)
BRIP1	2 (2.1)	10 (0.8)
ATR	10 (10.3)	38 (3.2)
Total (non-overlapping)	20 (20.6)	74 (6.1)
PI3K/AKT/mTOR pathway		
PIK3R1	6 (6.2)	18 (1.5)
PTEN	8 (8.2)	54 (4.5)
AKT1	8 (8.2)	58 (4.8)
Total (non-overlapping)	19 (19.6)	127 (10.5)
Receptor tyrosine kinases		
EGFR	6 (6.2)	13 (1.1)
ERBB2	7 (7.2)	33 (2.7)
ERBB3	6 (6.2)	29 (2.4)
Total (non-overlapping)	16 (16.5)	72 (6)

**Table 6:** Characteristics of the high and low mutation count groups in patients with ER-positive/HER2-negative breast cancer patients from DFCI. DCIS—ductal carcinoma in situ; IDC—invasive ductal carcinoma; ILC—invasive lobular carcinoma. The sum of the patients in each characteristic category is variable because of missing data.

Characteristic	Whole Group	High Mutation Count	Low Mutation Count	*p*
Age at metastasis (mean ± SD)	53.8 ± 11.2	55.1 ± 10.9	53.6 ± 11.2	0.4
Stage at diagnosis				
DCIS	2 (0.4)	1 (2.3)	1 (0.2)	0.3
I	75 (15.6)	6 (14)	69 (15.8)	
II	169 (35.3)	14 (32.6)	155 (35.6)	
III	111 (23.2)	9 (20.9)	102 (23.4)	
IV	122 (25.5)	13 (30.2)	109 (25)	
Histology				
IDC	322 (67.9)	29 (67.4)	293 (67.2)	0.11
ILC	83 (17.5)	8 (18.6)	75 (17.2)	
Mixed	60 (12.7)	5 (11.6)	55 (12.7)	
Other	9 (1.9)	1 (2.3)	8 (1.8)	
Grade				
I	44 (9.6)	2 (4.7)	42 (9.6)	0.58
II	246 (53.5)	22 (51.2)	224 (51.4)	
III	170 (36.9)	16 (37.2)	154 (35.3)	
Time to recurrence (months ± SD)		76.7 ± 44.2	65.2 ± 45	0.18
Recurrence				
Delayed (>5 years)	170 (47.6)	17 (56.7)	153 (46.8)	0.34
Early (<5 years)	187 (52.4)	13 (43.3)	174 (53.2)	
Subtype at metastasis diagnosis				
ER+/HER2−	366 (76.4)	33 (76.7)	333 (76.4)	0.32
Triple negative	30 (6.2)	4 (9.3)	26 (6)	
HER2+	9 (1.9)	2 (4.6)	7 (1.6)	
Unknown	74 (15.4)	4 (9.3)	70 (16.1)	
Treatment				
Adjuvant tamoxifen				
Yes	218 (45.5)	22 (51.2)	196 (45)	0.52
No	261 (54.5)	21 (48.8)	240 (55)	
Adjuvant aromatase inhibitor				
Yes	164 (34.5)	13 (30.2)	151 (34.6)	0.73
No	311 (65.5)	29 (67.4)	282 (64.7)	
(Neo)adjuvant chemotherapy				
Yes	288 (60.1)	23 (53.5)	265 (60.8)	0.41
No	191 (39.9)	20 (46.5)	171 (39.2)	

**Table 7:** Key characteristics and results of the ER-positive/HER2-negative/luminal breast cancer patients in the 3 series included in the current analysis. NA—not available.

	TCGA	METABRIC	DFCI
PIK3CA mutations	Higher prevalence in high TMB	Higher prevalence in high TMB	Higher prevalence in high TMB
TP53 mutations	Not statistically different	Higher prevalence in high TMB	Higher prevalence in high TMB
CDH1 mutations	Higher prevalence in high TMB	Higher prevalence in high TMB	Not statistically different
GATA3 mutations	Not statistically different	Not statistically different	Not statistically different
MMR mutations	Higher prevalence in high TMB	NA	Higher prevalence in high TMB
DDR mutations	Higher prevalence in high TMB	NA	Higher prevalence in high TMB
AS	Not statistically different	NA	NA
Survival	Not statistically different	Not statistically different	Not statistically different
